# Bridge-Enhanced Anterior Cruciate Ligament Repair Using Adjustable-Loop Cortical Suspensory Femoral Fixation

**DOI:** 10.1016/j.eats.2024.103048

**Published:** 2024-06-04

**Authors:** Thomas Y. Wu

**Affiliations:** Ventura County Medical Center, Ventura, California, U.S.A.

## Abstract

Presented here is a modified technique for bridge-enhanced anterior cruciate ligament repair using adjustable-loop cortical suspensory femoral fixation. Advantages include the elimination of the need for a larger femoral-side incision and elimination of the risk of knot slippage while securing fixation of the anterior cruciate ligament repair suture.

Bridge-enhanced anterior cruciate ligament repair (BEAR) has been shown to be a viable procedure with outcomes not inferior to autograft anterior cruciate ligament (ACL) reconstruction.^1^ It offers the significant advantage of avoiding harvest site morbidity compared with reconstruction with autograft. The original BEAR technique involves repair of the ACL to the femoral side using a No. 2 Vicryl (Ethicon) suture, tied down over a femoral cortical button, with 2 No. 2 Ethibond (Ethicon) sutures internally bracing the repair using the femoral button and an additional tibial cortical button.^2^

Modifications of the original technique in clinical practice have included use of high-strength sutures and arthroscopic passage of the suture grasping the ACL.^3^^,^^4^ Generally, a larger femoral-side incision of at least a couple of centimeters has been used for tying of the ACL repair suture on the femoral button through the distal thigh soft tissues, including the iliotibial band, to ensure that tension is not lost during the tying process.

The intent behind the technique presented here is to try to adhere to the mechanics of the original technique, which has demonstrated good outcomes, while taking advantage of more advanced suture materials and implant technology to result in stronger fixation while avoiding the need for a larger femoral-side incision and eliminating the risk of knot slippage while securing fixation of the ACL repair suture.

## Surgical Technique

Arthroscopy is performed in the supine position (Video 1). Anterolateral and anteromedial portals are established adjacent to the patellar tendon. Diagnostic arthroscopy is performed to confirm that there is sufficient ACL tissue attached to the tibia for repair. A far anteromedial portal is established low and medial, optimized for access to the femoral ACL footprint. Any necessary meniscal work is completed. Anterolateral notchplasty is perform if needed to improve visualization of the ACL femoral footprint with the arthroscope in the anterolateral portal.

**Fig 1:**
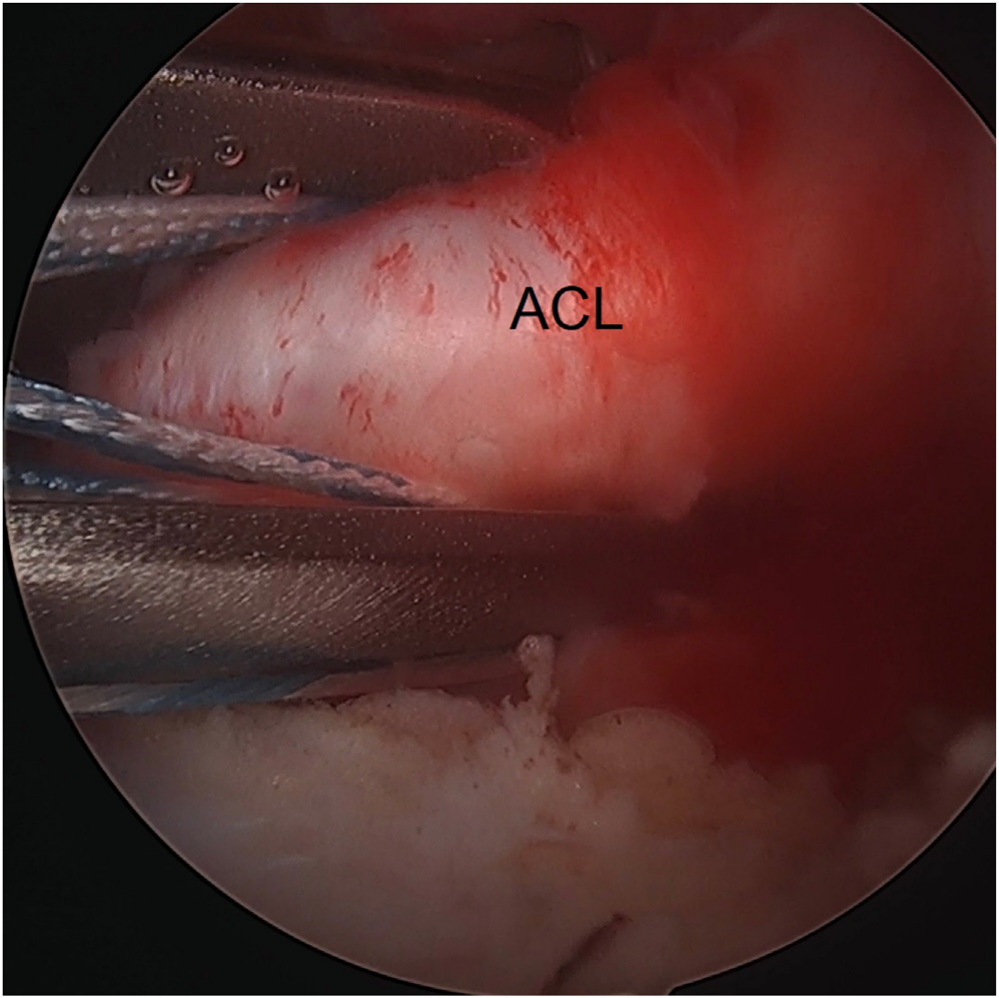
Left knee. Placement of the ACL-grasping suture. A 1.3-mm SutureTape (Arthrex) is passed in Bunnell fashion from distal to proximal in the ACL stump using a Scorpion suture passer. Care is taken to avoid cutting previously passed portions of the suture with the passer. A minimum of 1 transverse pass and 2 crossing oblique passes are done. (ACL, anterior cruciate ligament.)

An 8-mm × 3-cm PassPort cannula (Arthrex) is placed in the anteromedial portal to prevent soft tissue bridges. Through the anteromedial portal, a FastPass Scorpion SL suture passer (Arthrex) is used to pass 1.3-mm SutureTape (Arthrex) in Bunnell fashion from distal to proximal in the ACL stump (Fig 1). Care is taken to avoid cutting previously passed portions of the suture with the passer by keeping one limb more anterior and the other limb more posterior. A minimum of 1 transverse pass and 2 crossing oblique passes are done to grasp the ACL. The suture limbs are parked in the anteromedial portal.

**Fig 2:**
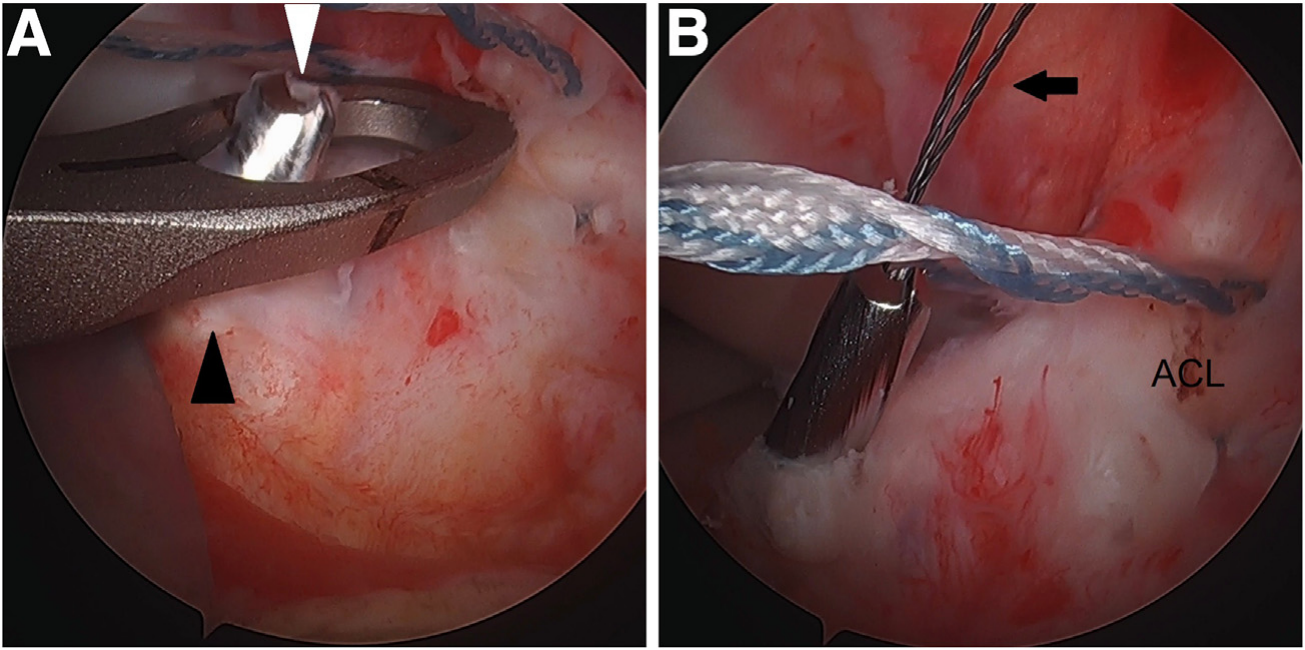
Left knee. Tibial tunnel creation. (A) Using an ACL tibial drill guide (black arrowhead) via the anteromedial portal, a 2.4-mm cannulated drill (white arrowhead) is drilled from the anteromedial proximal tibia to the anterior portion of the ACL tibial footprint. (B) A nitinol loop (black arrow) is passed up the cannulation of the drill, and the drill is removed. The nitinol loop can be switched out for a FiberLink (Arthrex) shuttling suture to minimize risk of breakage during shuttling of multiple sutures later. (ACL, anterior cruciate ligament.)

The tibial tunnel is drilled next. A 3- to 4-cm longitudinal incision is made on the anteromedial proximal leg for placement of an ACL tibial drill guide. Using the ACL tibial drill guide via the anteromedial portal, a 2.4-mm cannulated drill (Arthrex) is drilled from the anteromedial proximal tibia to the anterior portion of the ACL tibial footprint (Fig 2A). A nitinol loop is passed up the cannulation of the drill (Fig 2B), and the drill is removed. The nitinol loop can be switched out for a high-strength shuttling suture (FiberLink; Arthrex) to minimize risk of breakage during shuttling of multiple sutures later. It is parked in the anterolateral portal.

**Fig 3:**
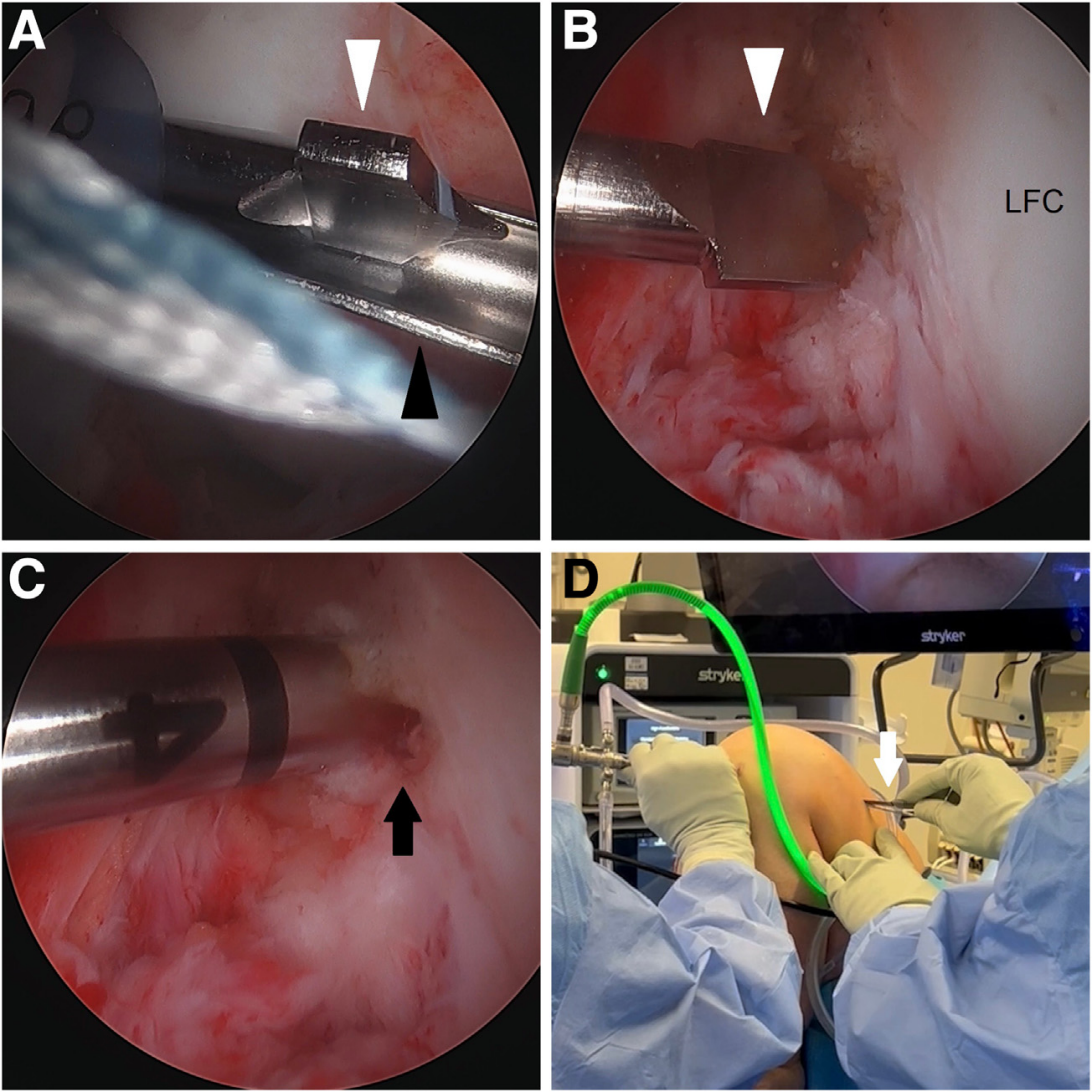
Left knee. Femoral tunnel creation. (A) With the medial femoral condyle protected by a half-pipe (black arrowhead), a spade-tipped Beath pin (white arrowhead) is placed through the far anteromedial portal. (B) With the knee in deep flexion, the pin (white arrowhead) is malleted to engage the tip in bone at the anterior portion of the ACL femoral footprint. (C) The pin is drilled out the anterolateral femoral cortex, and the intraosseous distance is measured using the pin (black arrow). (D) After the pin tip is driven out the skin, a No. 11 blade (white arrow) is used to slightly enlarge the pin site down through the iliotibial band to facilitate tying of a security knot later. The pin is then used to pass a TigerLink (Arthrex) shuttling suture. (ACL, anterior cruciate ligament; LFC, lateral femoral condyle.)

The femoral tunnel is drilled next. With the medial femoral condyle protected by a half-pipe, a spade-tipped Beath pin (4-mm ACL TightRope drill pin; Arthrex) is placed through the far anteromedial portal (Fig 3A). With the knee in deep flexion, the pin is malleted to engage the tip in bone at the anterior portion of the ACL femoral footprint (Fig 3B). The pin is drilled out the anterolateral femoral cortex. The intraosseous distance is measured using the pin (Fig 3C). The pin tip is then driven out the skin. A No. 11 blade is used to slightly enlarge the pin site down through the iliotibial band to facilitate tying of a security knot later (Fig 3D). The pin is used to pass a shuttling suture (TigerLink [Arthrex] for visual differentiation from the FiberLink).

**Fig 4:**
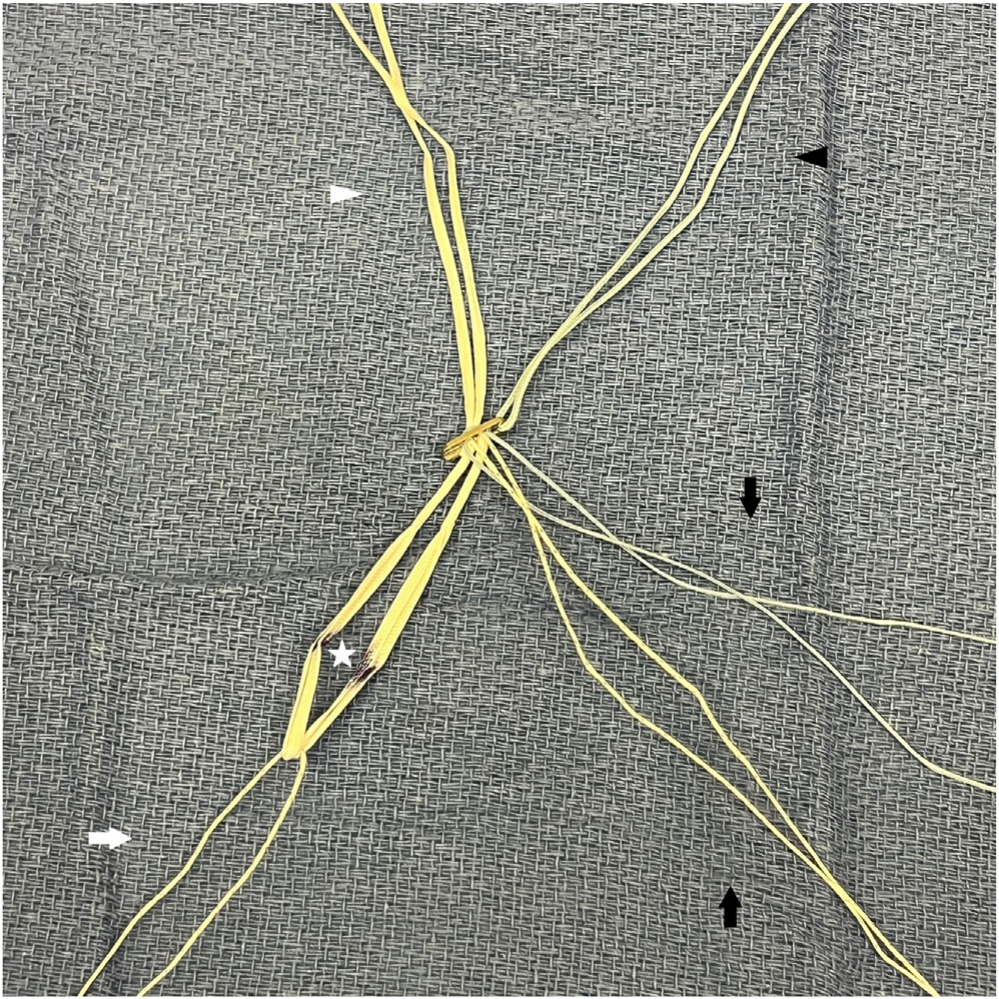
The adjustable-loop cortical suspensory femoral fixation (TightRope II RT; Arthrex) is prepared as follows. Two No. 2 high-strength sutures (black arrows) are looped through the 2 center holes of the button, with tails going distally along with the adjustable loop. These will be used for the internal brace. A No. 2 TigerWire (Arthrex) (white arrow) is placed through the adjustable loop, to be used for countertraction. The adjustable loop is marked at the measured intraosseous distance from the button (white star). The black arrowhead points to the leading FiberWire (Arthrex) of the implant. The white arrowhead points to the white tensioning sutures of the adjustable loop.

The adjustable-loop cortical suspensory femoral fixation (TightRope II RT; Arthrex) is prepared as follows (Fig 4). Two No. 2 high-strength sutures (FiberWire [Arthrex] and TigerWire [Arthrex] for visual differentiation) are looped through the 2 center holes of the button, with tails going distally along with the adjustable loop. These will be used for the internal brace. A No. 2 TigerWire is placed through the adjustable loop, to be used for countertraction. The adjustable loop is marked at the measured intraosseous distance from the button.

**Fig 5:**
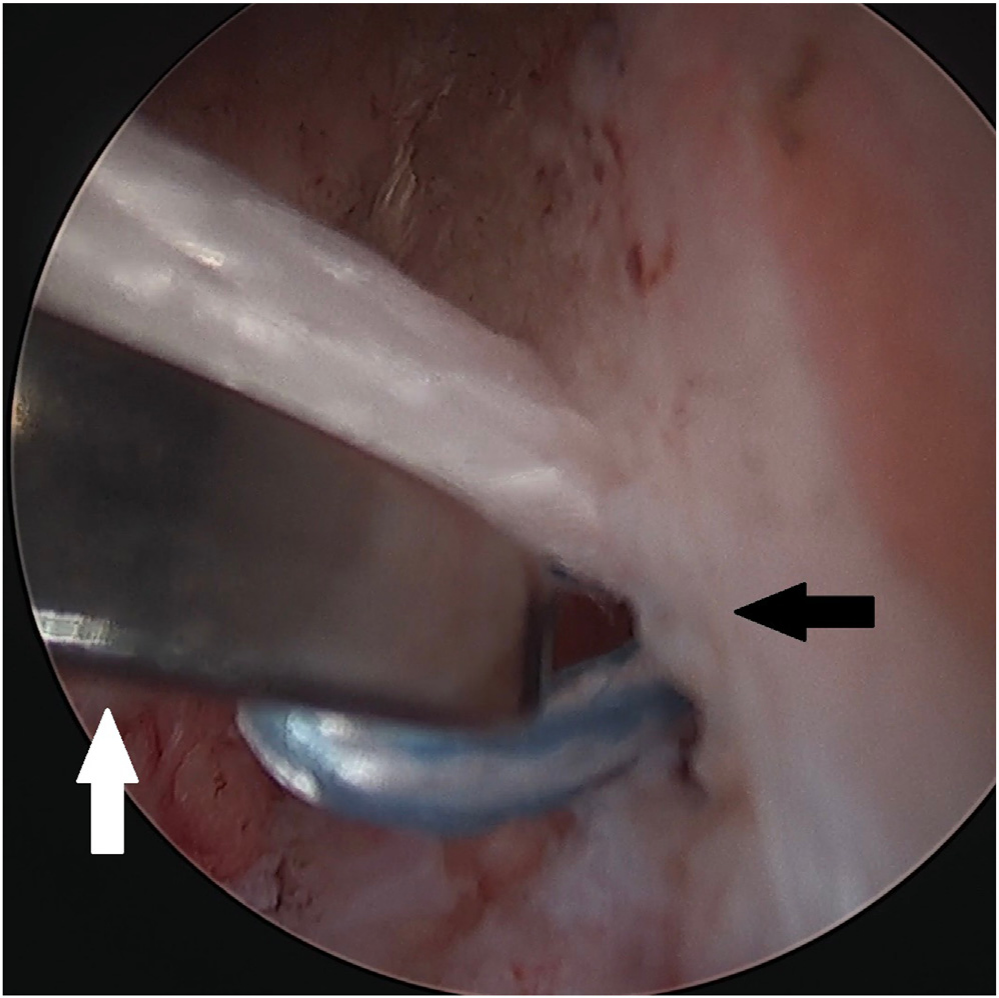
Left knee. Via the far anteromedial portal, the leading FiberWire (Arthrex) and the white tensioning sutures of the adjustable loop are shuttled through the femoral tunnel (black arrow). White arrow points to the suture button. Distal countertraction is provided using the TigerWire (Arthrex) in the adjustable loop. The button is flipped against the femoral cortex by pulling distally on the TigerWire in the adjustable loop after the intraosseous distance is reached.

Via the far anteromedial portal, the leading FiberWire and the white tensioning sutures of the adjustable loop are shuttled through the femoral tunnel (Fig 5). Distal countertraction is provided using the TigerWire in the adjustable loop. The button is flipped against the femoral cortex by pulling distally on the TigerWire in the adjustable loop after the intraosseous distance is reached. Button position can be checked using a C-arm.

**Fig 6:**
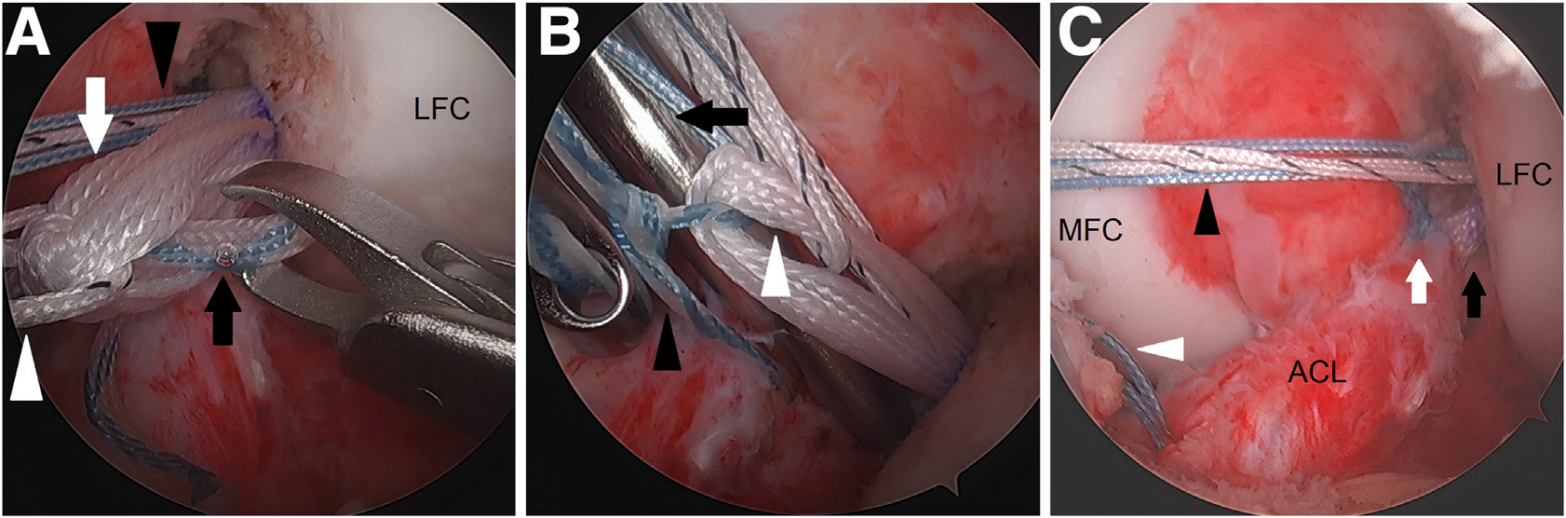
Left knee. The ACL-grasping suture is linked to the adjustable loop. (A) One limb of the ACL-grasping suture (black arrow) is passed through the adjustable loop (white arrow). Black arrowhead points to the internal brace sutures. White arrowhead points to the TigerWire (Arthrex) countertraction suture in the adjustable loop. (B) With a switching stick (black arrow) placed through the far anteromedial portal to prevent a tight capture that would prevent normal sliding of the adjustable loop, the ACL-grasping suture (black arrowhead) is tied to link it to the adjustable loop (white arrowhead). (C) The loop (black arrow) is shortened until most of the slack is taken out of the ACL-grasping suture (white arrow). The black arrowhead points to the internal brace sutures. The white arrowhead points to the tibial shuttling suture. (ACL, anterior cruciate ligament; LFC, lateral femoral condyle; MFC, medial femoral condyle.)

Next, the ACL-grasping suture is linked to the adjustable loop. With the arthroscope in the anteromedial portal, a crabclaw is used via the anterolateral portal to bring 1 limb of the ACL-grasping suture through the adjustable loop and pass it to a crabclaw to bring it out the far anteromedial portal (Fig 6A). The TigerWire countertraction suture in the adjustable loop helps identify the correct path for passage through the multiple strands of the adjustable loop. The arthroscope is moved to the anterolateral portal. The passed limb of the ACL-grasping suture is moved back into the anteromedial portal. With a switching stick placed through the far anteromedial portal to prevent a tight capture that would prevent normal sliding of the adjustable loop, the ACL-grasping suture is arthroscopically tied to link it to the adjustable loop (Fig 6B). While maintaining distal countertraction with the TigerWire in the adjustable loop, the loop is shortened by alternatingly pulling the white tensioning sutures until most of the slack is taken out of the ACL-grasping suture (Fig 6C). The TigerWire in the adjustable loop and the PassPort cannula are removed. The tibial shuttling suture is moved to the far anteromedial portal while ensuring no soft tissue bridge between it and internal brace sutures.

**Fig 7:**
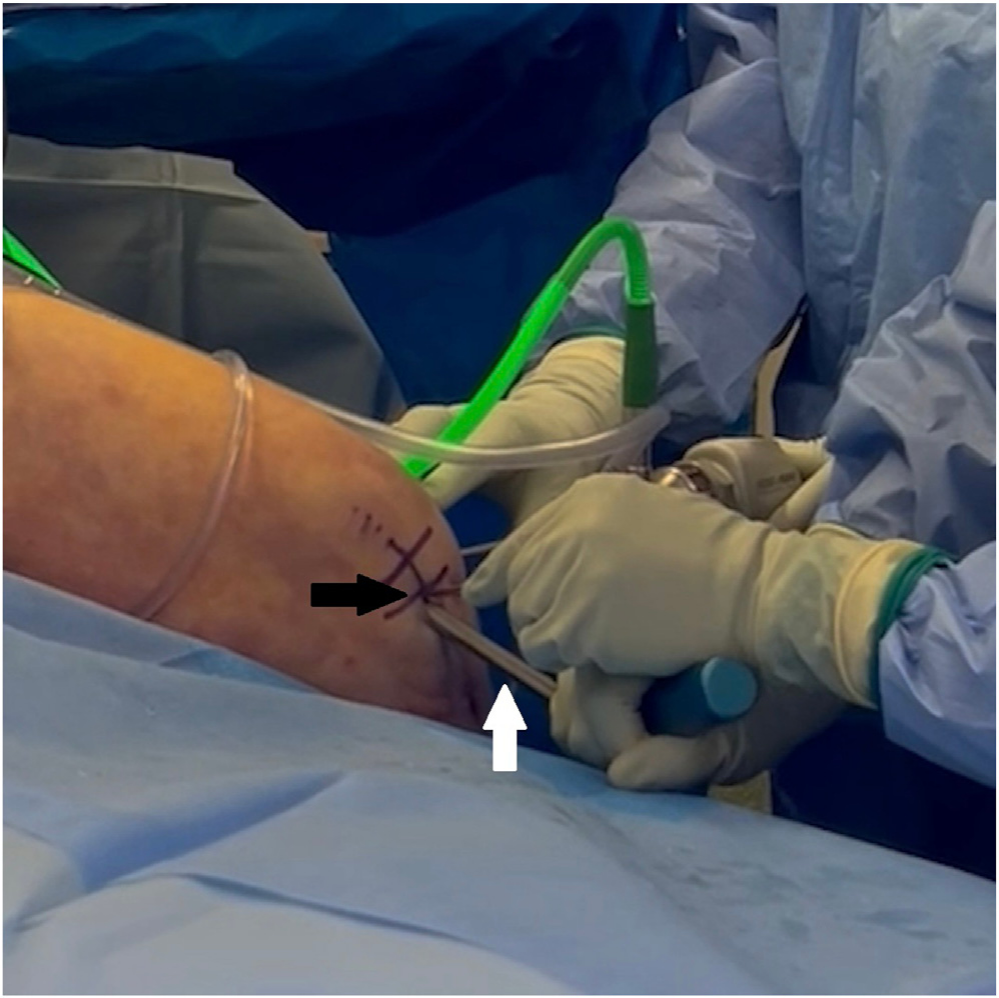
Left knee. Under arthroscopic visualization to avoid iatrogenic intra-articular injury, an arthrotomy of around 4 cm is made by proximally extending the far anteromedial portal (black arrow). A half-pipe (white arrow) is used to protect the sutures in the portal.

**Fig 8:**
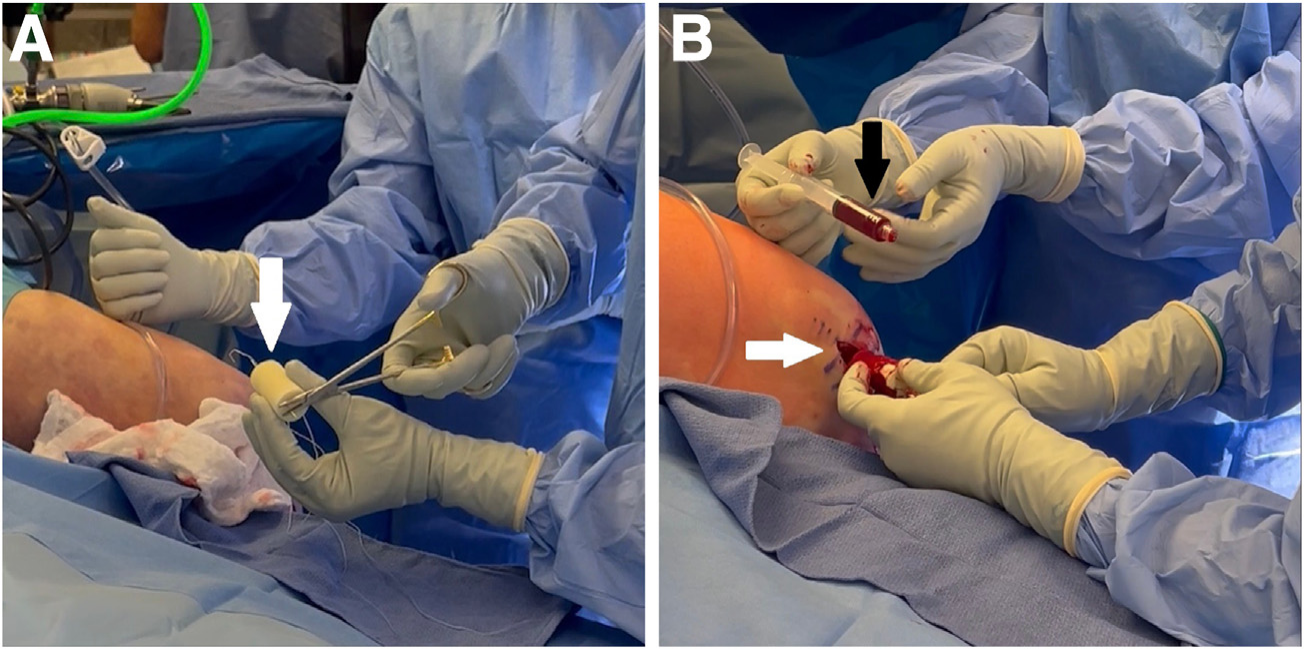
Left knee. Placement of the BEAR implant. (A) The 4 limbs of the 2 internal brace sutures are passed longitudinally through the BEAR implant (white arrow) using a straight needle. The 4 limbs are then shuttled down the tibial tunnel. (B) Then, 10 mL of fresh autologous blood (black arrow) is worked into the BEAR implant (white arrow), which is then pushed into the knee, guided by the internal brace sutures into the femoral notch. (BEAR, bridge-enhanced anterior cruciate ligament repair.)

**Fig 9:**
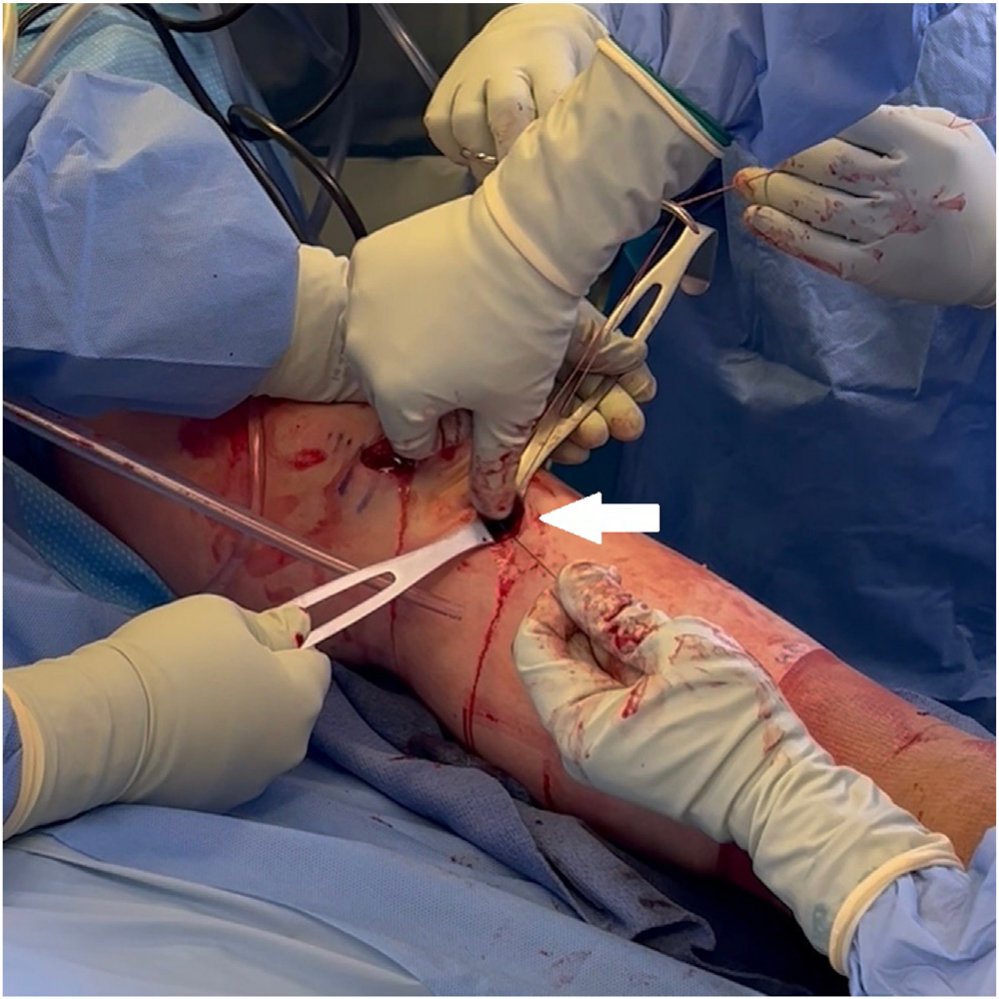
Left knee. The internal brace sutures are tied over a simple cortical button (white arrow) over the tibia with the knee in full extension.

Under arthroscopic visualization to avoid iatrogenic intra-articular injury, an arthrotomy of around 4 cm is made by proximally extending the far anteromedial portal. A half-pipe is used to protect the sutures in the portal (Fig 7). The knee is dried using sponges. Gloves are changed to dry ones. The 4 limbs of the 2 internal brace sutures are passed longitudinally through the BEAR implant (Miach Orthopaedics) from the porous end to the dense end using a straight needle (Fig 8A). Care is taken to avoid skewering previously placed suture limbs by checking that the previously passed limbs can still slide within the BEAR implant after the needle is placed for the next passage and before pulling the new limb through the implant. The 4 limbs are then shuttled down the tibial tunnel using the tibial shuttling suture. Then, 10 mL of fresh autologous blood is worked into the BEAR implant, starting with the proximal end and then the sides of the cylindrical implant. The distal end is left dry to maintain structural integrity to push against when inserting the implant into the knee. Before the implant is too soft, it is pushed into the knee, guided by the internal brace sutures into the femoral notch while tension is maintained on the sutures from outside the arthrotomy (Fig 8B). Once the implant is in place, traction is pulled on the internal brace sutures through the tibial tunnel distally to remove all slack. The internal brace sutures are tied over a simple cortical button (3.5 mm Suture Button; Arthrex) over the tibia with the knee in full extension (Fig 9). Adequate tensioning is ensured by doing 2 passes over and under with each suture, as in a surgeon’s knot, and then alternatingly tightening the sutures before finishing tying one while holding tension on the other.

**Fig 10:**
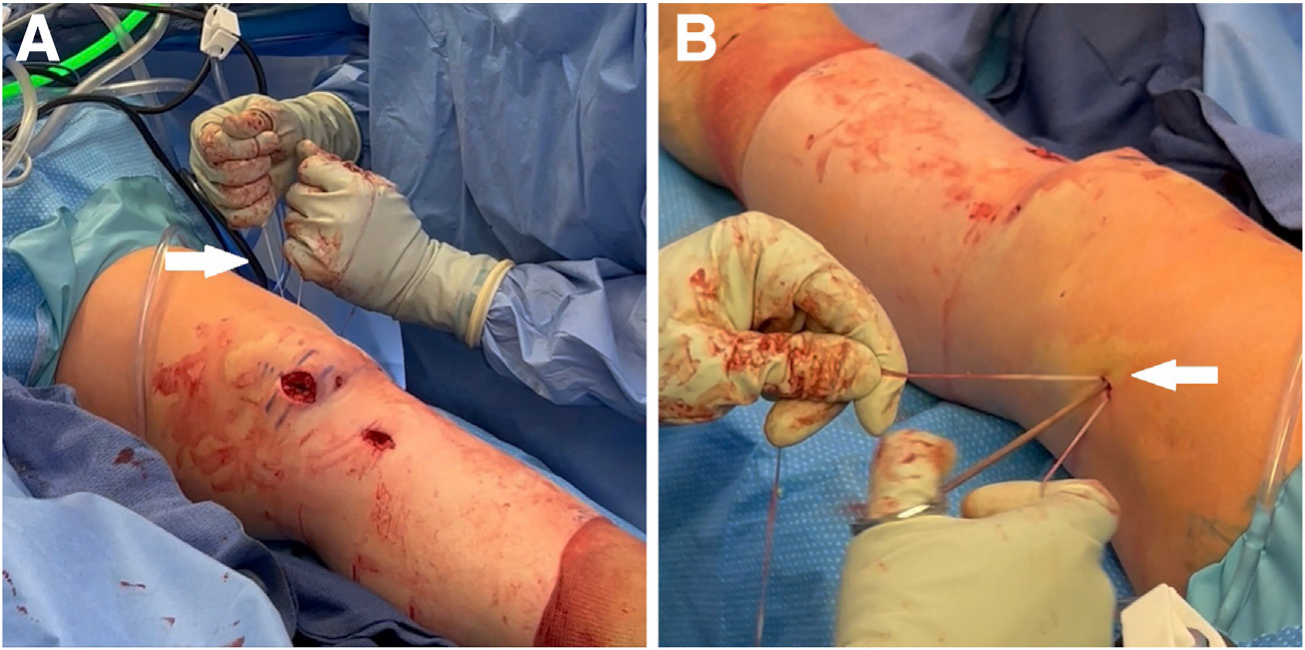
Left knee. (A) The adjustable loop is then used to take all remaining slack out of the ACL-grasping suture by alternatingly pulling the white tensioning sutures (white arrow). (B) For further security, the tensioning sutures of the adjustable loop are tied using a knot pusher (white arrow). (ACL, anterior cruciate ligament; BEAR, bridge-enhanced anterior cruciate ligament repair.)

The adjustable loop is then used to take all remaining slack out of the ACL-grasping suture by alternatingly pulling the white tensioning sutures (Fig 10A). For further security, the tensioning sutures of the adjustable loop are tied using a knot pusher (Fig 10B).

Routine closure is performed. The knee is braced in extension. Postoperative rehabilitation is as per standard protocol of the BEAR implant manufacturer available on the manufacturer’s website (Miach Orthopaedics).

Pearls and pitfalls of the procedure are listed in Table 1.

**Table 1:** Pearls and Pitfalls of the Technique

Pearls
• An anterolateral notchplasty can help improve visualization of the ACL femoral footprint.
• Place a PassPort (Arthrex) cannula in the anteromedial portal to prevent soft tissue bridges.
• After drilling the tibial tunnel, switching the nitinol loop out for a high-strength shuttling suture (FiberLink; Arthrex) helps to minimize risk of breakage during shuttling of multiple sutures later.
• Protect the medial femoral condyle with a half-pipe when inserting the spade-tipped Beath pin into the knee for drilling the femoral tunnel.
• Measuring the intraosseous distance of the femoral tunnel and marking it on the adjustable loop helps the surgeon to know when to flip the femoral button.
• The TigerWire (Arthrex) countertraction suture in the adjustable loop also helps identify the correct path through the multiple strands of the loop during arthroscopic passage of the ACL-grasping suture through the loop.
• Ensure that there is no soft tissue bridge between the tibial shuttling suture and the internal brace sutures by grasping all limbs with a crabclaw through the far anteromedial portal.
• Make sure that the knee and the gloves are dry before starting to work with the BEAR implant to avoid unintentional premature softening of the implant.
• When passing the internal brace sutures through the BEAR implant, avoid skewering previously placed suture limbs by checking that the previously passed limbs can still slide within the implant after the needle is placed for the next passage and before pulling the new limb through the implant.
• When tying the internal brace sutures over the cortical button over the tibia, adequate tensioning is ensured by doing 2 passes over and under with each suture, as in a surgeon’s knot, and then alternatingly tightening the sutures before finishing tying one while holding tension on the other.

Pitfalls
• When arthroscopically passing suture through the ACL, the needle in the passer can cut previously passed portions of the suture. Avoid this by keeping one suture limb more anterior and the other suture limb more posterior.
• If the BEAR implant is allowed to soften too much from the autologous blood, it loses its structural integrity and becomes difficult to push into the knee along the internal brace sutures.
• If the anteromedial arthrotomy is too small, it is difficult to push the BEAR implant into the knee during the short window after addition of the autologous blood and before it becomes too soft. The arthrotomy should be large enough to easily allow entry of the surgeon’s thumb.

ACL, anterior cruciate ligament; BEAR, bridge-enhanced anterior cruciate ligament repair.

## Discussion

The original BEAR technique requires the ACL repair suture to be tied down on the femoral cortical button. Many surgeons choose to do this through a larger incision to ensure that tension is not lost in the repair suture during the tying process. The key innovation in the technique presented here is use of an adjustable-loop cortical suspensory femoral fixation. This prevents loss of tension during securing of fixation, without the need for a larger femoral-side incision. The tensioning sutures of the adjustable loop are tied percutaneously at the end of the procedure to eliminate any risk of later slippage, and this is easily done through a percutaneous incision since tension is already maintained by the adjustable loop.

Two key concepts enable the use of the adjustable-loop cortical suspensory fixation in this application. First, the use of a temporary suture through the adjustable loop to provide countertraction provides full control of the implant during passage and shortening in the absence of a graft through the loop. Second, the use of a switching stick during tying of the ACL-grasping suture around the adjustable loop allows secure linkage without a tight capture that would prevent normal sliding of the adjustable loop during shortening.

The main advantages of this technique are the elimination of the need for a larger femoral-side incision and elimination of the risk of knot slippage while securing fixation of the ACL repair suture (Table 2). Additionally, the need for overdrilling the guide pin has been eliminated on both the femoral and tibial sides. Also, the use of high-strength sutures improves the strength of the construct. Limitations include higher implant costs and increased complexity compared with the original BEAR technique. Compared with ACL reconstruction, this technique has advantages and limitations similar to the original BEAR technique. Advantages include preservation of the native ligament, avoidance of harvest site morbidity, and avoidance of disease transmission in the case of reconstruction with allograft. Limitations include potential inability to perform the procedure due to inadequate ACL tissue for repair and risk of failure of repair.

**Table 2:** Advantages and Limitations of the Technique

Advantages
• Elimination of need for a larger femoral-side incision
• Elimination of risk of knot slippage while securing fixation of ACL repair suture
• Elimination of need for overdrilling guide pins
• Stronger construct
• Preservation of the native ligament compared with ACL reconstruction
• Avoidance of harvest site morbidity compared with ACL reconstruction with autograft
• Avoidance of disease transmission compared with ACL reconstruction with allograft

Limitations
• Higher implant costs
• Higher complexity
• Potential inability to perform the procedure due to inadequate ACL tissue for repair
• Risk of failure of repair

ACL, anterior cruciate ligament.

## Disclosures

The author declares that they have no known competing financial interests or personal relationships that could have appeared to influence the work reported in this paper.

## Supplementary Data

Video 1 This is a technique video for bridge-enhanced anterior cruciate ligament repair (BEAR) using adjustable-loop cortical suspensory femoral fixation. Left knee. To grasp the anterior cruciate ligament (ACL), 1.3-mm SutureTape (Arthrex) is passed in Bunnell fashion from distal to proximal in the ACL stump. A 2.4-mm cannulated drill is drilled from the anteromedial proximal tibia to the anterior portion of the ACL tibial footprint and used to pass a shuttling suture. With the knee in deep flexion, a spade-tipped Beath pin through the far anteromedial portal is driven into the anterior portion of the ACL femoral footprint and out the anterolateral femoral cortex. The pin is used to pass a shuttling suture. The adjustable-loop cortical suspensory femoral fixation (TightRope II RT; Arthrex) is prepared as follows. Two No. 2 high-strength sutures are looped through the 2 center holes of the button, with tails going distally along with the adjustable loop, to be used for the internal brace. A temporary suture is placed through the adjustable loop, to be used for countertraction. The button is shuttled through the femoral tunnel while distal countertraction is provided using the temporary suture in the adjustable loop. The button is flipped against the femoral cortex. The ACL-grasping suture is securely linked to the adjustable loop while avoiding a tight capture that would prevent normal sliding of the adjustable loop. An arthrotomy is made by proximally extending the far anteromedial portal. The 4 limbs of the 2 internal brace sutures are passed longitudinally through the BEAR implant and then shuttled down the tibial tunnel. Then, 10 mL of fresh autologous blood is worked into the BEAR implant. The implant is pushed into the knee, guided by the internal brace sutures into the femoral notch. The sutures are tied over a simple cortical button with the knee in full extension. The adjustable loop is used to take all remaining slack out of the ACL-grasping suture. For further security, the tensioning sutures of the adjustable loop are tied using a knot pusher.
